# Factors Affecting Eating Motivation Play a Role in Orthorexia Nervosa in University Students: A Cross-Sectional Study

**DOI:** 10.3390/bs15030301

**Published:** 2025-03-04

**Authors:** Rabia Melda Karaağaç, Indrani Kalkan

**Affiliations:** Department of Nutrition and Dietetics, Institute of Health Sciences, Istanbul Medipol University, Beykoz, Istanbul 34810, Turkey; indrani.kalkan@medipol.edu.tr

**Keywords:** eating motivation, orthorexia nervosa, eating disorders, health, university students

## Abstract

Orthorexia nervosa (ON), an eating disorder marked by an obsession with healthy eating, is influenced by social and psychological factors, particularly among university students during a formative period. This study investigated the effects of eating motivations, defined by factors such as health, pleasure, social influences, and environmental concerns, on orthorexic tendencies. Using the Turkish-validated versions of ORTO-11 and The Eating Motivation Survey (TEMS—Brief version), data were collected from 416 students who meet the study participation criteria (mean age: 22.3 ± 4.41 years, 84.4% female, and mean BMI: 22.74 ± 4.54 kg/m^2^). The results showed that ORTO-11 scores increased significantly with BMI, indicating lower orthorexic tendencies. TEMS sub-dimensions revealed that the health sub-dimension decreased orthorexic tendencies (*p* = 0.044), whereas the traditional eating sub-dimension significantly increased them (*p* = 0.000). These findings suggest a complex interaction where prioritizing health may conflict with cultural eating norms. Interventions should address this balance by promoting a holistic approach to nutrition, integrating stress management techniques, and raising awareness of ON through targeted workshops and educational programs for students and health professionals. A long-term evaluation of these programs is essential to ensure their effectiveness in fostering healthier relationships with food and mitigating ON risk among young adults.

## 1. Introduction

Orthorexia nervosa (ON) is defined as an excessive obsession with eating healthy or “pure” foods. Although ON shares certain overlapping characteristics, it is distinct from other eating disorders, such as anorexia nervosa and conditions like obsessive–compulsive disorder (OCD) (Horovitz & Argyrides, 2023; Gkiouleka et al., 2022). Unlike anorexia nervosa, which is primarily characterized by a desire to lose weight and a fear of gaining weight, ON is driven by an obsession with food quality and purity rather than quantity (Dunn & Bratman, 2016). Similarly, while ON shares traits with OCD, such as compulsive behaviors and perfectionism, it is specifically focused on dietary habits rather than a broader range of obsessive behaviors (Koven & Abry, 2015). In the scientific literature, a distinction is often made between ON and “healthy orthorexia”. Healthy orthorexia refers to a balanced and health-conscious approach to eating, where individuals aim to maintain a nutritious diet without excessive rigidity or negative psychological impacts. In contrast, ON is characterized by an obsessive focus on food quality and purity, leading to restrictive eating patterns and potential impairment in social and psychological functioning (Dunn & Bratman, 2016). Recognizing this distinction is essential for understanding the continuum of orthorexic behaviors and for identifying individuals at risk of developing ON (Strahler, 2020). Although not formally recognized as a diagnostic category, ON is increasingly acknowledged within mental health research, including eating behaviors that can disrupt daily life, harm social relationships, and also lead to malnutrition in some cases.

ON has gained increasing attention in the last decade, with a growing body of research focusing on its psychological and behavioral aspects (Missirio et al., 2021). Adolescents and young adults are among the vulnerable groups, not only because of their rapid developmental transitions but also their psychological changes in the context of decision making, choices, preferences, belief systems, etc. (Hardgrove, 2014). It is also a period of increased energy and nutrient requirements, necessary to meet the increasing demands of developmental well-being, which in turn promote optimal physical, cognitive and mental health (Barnes & Caltabiano, 2017). Furthermore, this age group is sensitive to negative body image, low self-esteem, weight pressure, and disordered eating behaviors such as anorexia and bulimia (which may or may not be associated with different forms of malnutrition such as undernutrition, obesity, and deficiencies of specific nutrients (World Health Organization, 2005; Christian & Smith, 2018), making it a challenging time for a person’s nutrition and adequate nutrient intake. The prevalence of ON risk has been shown to be as high as 20.4% among university students, rising between 35.0 and 57.8% for people belonging to high-risk groups including nutritionists, students of nutrition, medicine, nursing and other healthcare-related professions, fitness and yoga instructors, gymnasts and trainers, and even dancers and young artists (Gkiouleka et al., 2022). Adolescence and young adults are critical developmental stages where individuals are particularly vulnerable to disordered eating behaviors. The psychological changes that occur during these periods, such as identity development, need for social approval, perfectionism, and increased stress levels, may contribute to the onset ON. These factors can drive an excessive focus on healthy eating, which may evolve into pathological behaviors (Rodgers et al., 2013). Additionally, the influence of social media and the widespread promotion of diet culture further exacerbate this tendency (Holland & Tiggemann, 2016).

Young adults may be vulnerable to ON due to factors such as social media use and peer pressure. The risk of ON is particularly high among university students and is linked to factors such as dietary awareness, body perception, stress levels, and social media use. For instance, social media promotes unrealistic health and beauty standards, leading to an excessive focus on food quality (Holland & Tiggemann, 2016; Cena et al., 2019). Additionally, peer pressure in academic or social settings can normalize restrictive dietary practices, increasing ON risk (Turner & Lefevre, 2017). Addressing these factors is crucial for preventing ON. A study by Villa et al. (2022) found that social media use is a factor that increases the risk of ON. The study showed that health and nutrition content shared on social media platforms can encourage restrictive dietary habits, with individuals exposed to such content tending to adopt overly restrictive eating behaviors. These findings support the idea that social media use may be a contributing factor to the increased risk of ON among young adults.

Eating motivation is the combination of biological, psychological, and social factors that shape an individual’s eating behavior. Biological aspects such as hunger and satiety cues form its foundation, while psychological elements like stress, emotional state, and body image concerns strengthen this motivation. Additionally, cultural influences, including social media and diet trends, significantly affect food choices and eating patterns. This motivation plays a crucial role in maintaining a healthy diet and can also contribute to the development of eating disorders (Gkiouleka et al., 2022; Stroebele & De Castro, 2004). For example, individuals with ON may avoid emotional or environmental eating triggers by maintaining strict control over their diets (Barnes & Caltabiano, 2017). ON and muscle dysmorphia (MD) are growing concerns among young adults. A study on Italian university students identified traits of both disorders, linking ON and MD, with excessive focus on eating and muscle development contributing to their prevalence (Gorrasi et al., 2020). The study also found that students from different academic disciplines exhibited varying tendencies toward orthorexia, emphasizing the influence of educational context on health behaviors (Guglielmetti et al., 2022). These findings highlight how biological, psychological, and social factors shape eating motivations and contribute to ON development, with social media and societal health perceptions further reinforcing these behaviors. Further research into these dynamics will deepen our understanding of ON.

As the awareness has increased regarding the growth of chronic diseases especially in the western world, over the last two decades, there has been an increasing focus on healthy eating and being slim, and having a fit body has become increasingly important. In this context, ON is not merely characterized by a general obsession with healthy eating but rather by an extreme and rigid focus on certain foods perceived as “healthy” and the exclusion of others deemed “unhealthy”. This distinction is critical, as the behaviors associated with ON go beyond health-conscious eating and instead reflect a restrictive and inflexible dietary pattern that may lead to nutritional imbalances and psychological distress (Kiss-Leizer et al., 2019). In individuals who self-identify as having orthorexia, participants report that chronic disease management leads to obsessions with healthy eating (Valente et al., 2020).

Understanding why we eat and the motivational factors that guide our food choices is very important in terms of assessing chronic diseases such as obesity, diabetes, and cardiovascular disease (Grimm & Steinle, 2011). The primary aim of this study is to explore the impact of factors influencing eating motivation on ON tendencies among university students. By examining these factors, the study seeks to contribute to a deeper understanding of the relationship between eating motivation and ON, particularly in young adults.

## 2. Materials and Methods

### 2.1. Study Design, Settings, and Participants

**Figure 1:**
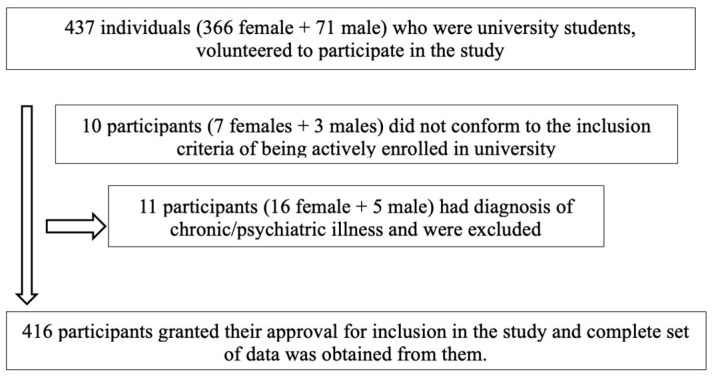
Flowchart showing participant recruitment process.

This cross-sectional study was conducted between February and April 2023 with 416 university students using an online survey database (Google Forms). Participants were recruited using random sampling. The participant recruitment process has been provided in Figure 1. The inclusion criteria for participants in the study was to be actively enrolled in the university as a graduate or undergraduate student and to be able to understand and express themselves in Turkish. A diagnosed chronic, psychiatric illness, or eating disorder was an exclusion criteria. Participants with chronic psychiatric illnesses or eating disorders were excluded to prevent potential confounding effects on eating motivation and ON, ensuring a more accurate assessment of factors related to ON. Due to a diagnosed psychiatric illness, the following 10 participants were excluded from the study. Participants with a history of eating disorders but currently in remission were excluded.

This study was approved by University Human Research Ethics Committee (No: 2023/01, 17 January 2023). Informed consent forms were obtained from all adults who volunteered to participate in the study. Participants were fully informed of the study’s specific objectives, potential risks, and the voluntary nature of their participation prior to providing informed consent. They were assured that participation was entirely voluntary and that they could withdraw at any time without consequence. Participants were not compensated or incentivized for their participation. However, the study ensured that all ethical standards were met to maintain the integrity of the research while encouraging voluntary participation. This study was conducted in accordance with the principles of the Declaration of Helsinki (World Medical Association, 2013).

### 2.2. Data Collection Tools and Evaluation

#### 2.2.1. Sociodemographic Information Form

This part of the questionnaire was developed by the researchers through a review of the literature. It consists of 12 items asking for demographic information such as age, gender, marital status, educational level, body weight, height, level of physical activity, and satisfaction with body weight.

#### 2.2.2. The Eating Motivation Survey (TEMS) Brief Form

The original TEMS (The Eating Motivation Survey) tool, a 7-point Likert scale comprising of 45 items, designed by Renner et al. (2012) is a comprehensive and multi-faceted measure of reasons for eating and making food choices. The Turkish adaptation of the scale was conducted by Karaağaç and Andaç Öztürk (2022). All of the 45 items of the original scale could be validated in Turkish, and the validated scale type also had a 7-point Likert structure. The sub-dimensions of TEMS are as follows: Liking, Habits, Need and Hunger, Health, Convenience, Pleasure, Traditional Eating, Natural Concern, Sociability, Price, Visual Appeal, Weight Control, Affect Regulation, Social Norms, and Social Image. The form’s focus on health-related and restrictive eating behaviors aligns with the key characteristics of ON, making it a relevant tool for understanding the motivations underlying this condition. All sub-dimensions of the TEMS may be related to ON. However, we hypothesized that especially the sub-dimensions of health, concern for nature and weight control might be directly related to ON.

#### 2.2.3. ORTO-11

The original scale (ORTO-15) was developed to measure the degree of obsession with healthy eating and comprised of 15 items (Donini et al., 2005). However, Arusoğlu et al. (2008) adapted and validated the ORTO-15 tool into Turkish. Following factor analyses, four items (1, 2, 9, and 15) with low statistical power were removed from the scale and validated as ORTO-11. ORTO-11, a 4-point Likert scale, is scored as always = 1, often = 2, sometimes = 3, and never = 4 points. Only item 8 is scored in the opposite direction. The cut-off point for this scale was set at ≤27. Individuals with a total score of 27 and below are defined as “ON-tendent” or hypersensitive, and, as the score increased, the eating behavior was considered to approach a normal level. The cut-off score of ≤27 was selected based on previous research in the Turkish context, where this threshold has been shown to effectively distinguish individuals with higher risk for ON. The cut-off score of ≤27 has been validated in previous studies, including those with university students and the Turkish population (Arusoğlu et al., 2008; Demir & Aşcı, 2019). To assess orthorexia nervosa, the ORTO-11 scale was developed and adapted into Turkish. The Turkish version of the scale has been shown to be a reliable measurement tool in validity and reliability studies conducted in Turkey. For example, in the Turkish adaptation of the Teruel Orthorexia Scale, the internal consistency coefficient of the ORTO-11 was found to be 0.77. This supports the reliability of the scale (Asarkaya & Arcan, 2020).

A review by Alshaibani et al. (2024) analyzed 21 different studies that had previously used the ORTO-11 scale and reported a summary Cronbach’s alpha value of 0.59 from these studies involving a total of 11,000 participants. In addition, Cronbach’s alpha values of individual studies were reported to vary between 0.23 and 0.83. The Cronbach’s alpha value obtained in this study was found to be 0.626, which is similar to the range reported in the aforementioned study.

### 2.3. Statistical Analysis

The data were analyzed using SPSS 22.0 software. The Shapiro–Wilk test for normality indicated that the data did not have a normal distribution (skewness kurtosis in the range of −1.5–+1.5; *p* < 0.05. No skewness or kurtosis values were found between +1.5 and −1.5, and it was determined that the data were not normally distributed. Therefore, analyses were performed using non-parametric tests. Number (*n*) and percentage values for categorical analysis, minimum, maximum, mean, standard deviation, and median were calculated for quantitative variables. The Mann–Whitney U test was used to compare paired groups, and the Kruskal–Wallis test was used to compare more than two groups. The Mann–Whitney U test was preferred as the appropriate option for pairwise group comparisons because the data did not show a normal distribution. The Kruskal–Wallis test was chosen because it is a common non-parametric method used when the normality assumption is violated when comparing the medians of more than one group. Spearman correlation test was conducted for correlation analysis and multiple linear regression test for effect analysis. Spearman correlation was used to examine the relationship between BMI (a continuous variable), TEMS subscales (ordinal variables), and ORTO-11 scores (a continuous variable). BMI, TEMS subscales, and ORTO-11 scores were included as independent variables in the regression model. These variables were chosen because of their potential effects as reported in the literature. The model controlled for potential confounders such as age, sex, and the level of physical activity. In the analyses, *p* < 0.05 was considered significant. However, the Bonferroni correction was applied to reduce the risk of type I error due to multiple comparisons. Also, Body Mass Index (BMI) was also calculated from the participants’ self-reported height and weight. According to the 80% power analysis, the minimum sample size was set at 343 subjects. In the sample size calculation, the type I error (α) was set at 0.05, and the effect size was set at the medium level (0.15) according to the linear regression analysis determined by Cohen (1988). G-Power (version 3.1.9.4) was used to calculate the power analysis. The initial sample size was larger, but, after applying the inclusion and exclusion criteria, the final number of participants that remained was 416. The expected dropout rate was considered in the power analysis, and the sample size was adjusted accordingly to ensure sufficient power for the study.

## 3. Results

The majority of participants who voluntarily agreed to take part in the study were females. It was found that 84.4% of the participants were female and 15.6% male, 91.8% were single, 80% were university graduates, 45.9% performed less than 150 min of physical activity per week, and 55.5% were not satisfied with their current weight. The mean age of the participants was 22.3 ± 4.41 years, the mean body weight was 63.24 ± 14.87 kg, the mean height was 1.66 ± 0.07 m, the mean BMI was 22.74 ± 4.54 kg/m^2^, and the mean score of the ORTO-11 was 27.55 ± 4.71 (Table 1). Also, 13.9% of the participants were underweight, 60.8% were of normal weight, 18.5% were overweight, and 6.8% were obese. No significant difference was found in BMI comparison between tendency to ON and no tendency to ON groups (X^2^: 6.301, *p* = 0.278).

The Cronbach alpha values of the scales used in the study were TEMS: 0.954 and ORTO-11: 0.626. A statistically significant difference was found between sub-dimension “satisfaction with current body weight” when comparing the ORTO-11 scale with the descriptive variables of participants with and without ON tendency (*p* < 0.05). This may indicate that people who are satisfied with their body weight are more likely to engage in healthy eating (orthorexia). This focus on healthy eating may be related to body weight satisfaction. In our study, sex and educational level showed a tendency toward statistical significance. We believe this finding may be related to the sample size, imbalances between groups, or indirect effects of these factors. Also, when comparing the orthorectic and non-orthorectic status of participants who did less than 150 min of physical activity per week according to their satisfaction with their current body weight, the rate of those who were satisfied with their body weight was significantly higher in orthorectics (53.1%) than in non-orthorectics (28%). The proportion of those who were dissatisfied with their body weight was significantly higher in non-orthorectic subjects (72%) than in orthorectic subjects (46.9%). A statistically significant relationship was found between body weight satisfaction and orthorexia tendency (*p* < 0.001), but the effect size of this relationship was found to be small according to Cohen’s guidelines (1988) (Table 2).

In the correlation analysis of the quantitative variables with the sub-dimensions of the TEMS, a statistically significant relationship was found between the body weight of the participants and weight control, affect regulation, and social image sub-dimensions; the height of the participants and health, traditional eating, and weight control sub-dimensions; BMI and weight control and affect regulation sub-dimensions at positive and low levels (*p* < 0.05) (Table 3). In our study, the classification of correlation strength specific to health sciences, as defined by Mukaka (2012), was applied. According to this classification, the strength of the relationship is categorized as follows: very weak (0.00 ≤ |r| < 0.20), weak (0.20 ≤ |r| < 0.40), intermediate (0.40 ≤ |r| < 0.60), strong (0.60 ≤ |r| < 0.80), and very strong (0.80 ≤ |r| ≤ 1) (Mukaka, 2012).

In the correlational analysis of the mean ORTO-11 scores of the ON-tendent participants and sub-dimensions of the TEMS, a positive low-level significant relationship was found between the mean ORTO-11 scores of the participants and the health and weight control sub-dimensions, whereas a negative, low-level significant relationship was found with the traditional eating sub-dimension (*p* < 0.05) (Table 4).

In the effect analysis of the quantitative variables related to the mean ORTO-11 scores of the ON-tendent participants, it was found that the model was adequate (F: 4.832; *p*: 0.003), the quantitative variables influenced orthorexia nervosa by 6.7% (R^2^: 0.067), and only the BMI variable had a positive and statistically significant effect (*p* < 0.05). If the effect size of BMI in the model is interpreted as R^2^ = 0.067, it shows a weak effect. According to the Cohen’s R^2^ interpretation, small effect R^2^ = 0.02, medium effect: R^2^ = 0.13, and large effect: R^2^ = 0.26. The effect analysis of the TEMS dimensions related to the mean ORTO-11 scores of the ON-tendent participants showed that the model was adequate (F: 5.788; *p*: 0.001), and the TEMS dimensions influenced orthorexia nervosa by 7.9% (R^2^: 0.079), with the health dimension positively and the traditional eating dimension negatively (*p* < 0.05). (Table 5).

## 4. Discussion

ON is defined as an obsessive preoccupation with healthy eating (Dunn & Bratman, 2016). Although there may be various causes for orthorexia nervosa in individuals, factors that influence eating motivation may also play a role. This study aimed to determine the effect of factors affecting eating motivation on ON in university students.

This study, designed as a cross-sectional research type, was conducted on 416 university students. The mean score of the ORTO-11 of the participants was 27.55 ± 4.71, the cut-off point being (≤27). Individuals with ORTO-11 scores of >27 were considered having no tendency towards ON. In this study, 49.5% of the participants were found to have ON tendency.

On comparing the ORTO-11 according to descriptive variables, no significant relationship was found between sex, marital status, the level of education, the presence of chronic diseases, smoking and alcohol consumption, and weekly physical activity (*p* > 0.05). Some studies have reported no significant difference between sex and ON tendencies, whereas some others have found ORTO-11 scores to be higher in females (Donini et al., 2005; Dege & Alphan, 2021; Fidan et al., 2010). Although there are inconsistent findings in terms of sex, the fact that the number of male participants in this study was significantly lower may have rendered the ORTO-11 scores to be non-significant between sexes. The imbalance in gender distribution, with fewer male participants, may have influenced the findings related to gender and ON tendencies. A more balanced sample, with an equal number of male and female participants, could yield more precise insights into potential gender differences in ON. Future research should aim to recruit a more representative gender distribution to better explore these dynamics.

In this study, no significant difference was found between smoking, alcohol consumption, and marital status and ON tendency (*p* > 0.05). In a similar study, although smoking and alcohol consumption did not affect ON tendency (*p* > 0.05), a significant relationship was found with marital status. ON symptoms were found to be more common among married students (*p* = 0.007) (Basaran & Ozbek, 2023). The relationship between marital status and eating behaviors, including ON tendencies, could be influenced by family-related health pressures or social dynamics. For instance, married individuals may prioritize health due to shared family responsibilities or societal expectations. Future research should examine these factors in more detail to better understand how marital status contributes to ON tendencies.

In this study, when comparing ORTO-11 scores according to descriptive variables, a statistically significant difference was found only in the sub-dimension “satisfaction with current body weight” for participants without ON tendency (*p* < 0.05). While 45.6% of the students without ON tendency were not satisfied with their current body weight, 54.4% were satisfied with their current body weight. Similarly, in another study with Nursing students in Turkey, 44.7% of students without ON tendency were concerned about being overweight, while 55.3% were not concerned (*p* < 0.05) (Duran, 2022).

In this study, in the relational analysis of quantitative variables with the dimensions of the TEMS, a statistically significant positive relationship was found between the BMI and the “weight control” and “affect regulation” sub-dimensions (*p* < 0.05). A study conducted by Sproesser et al. (2019) reported similar findings and found that individuals with higher BMI had significantly higher mean values for “weight control” and “affect regulation” sub-dimensions. Therefore, it may be considered that individuals with increased BMI may have higher weight control motivation. However, anticipating their ON tendency based on these emotions may be complex because in this study ORTO-11 scores increased in individuals with higher BMI (*p* < 0.05). In other words, orthorectic tendency decreased with increased BMI. In contrary, some studies have reported orthorexic tendency to have increased with higher BMI (Fidan et al., 2010; Oberle et al., 2017; Asil & Sürücüoğlu, 2015; Bundros et al., 2016). The unexpected negative association between higher BMI and ON tendencies observed in our study may be influenced by various cultural, psychological, or physical factors. For instance, cultural norms in Turkey might emphasize different eating behaviors among individuals with higher BMI, or psychological traits such as perfectionism and control might differ across BMI categories. Previous research has suggested that individuals with lower BMI may be more likely to exhibit orthorexic tendencies due to a stronger focus on dietary control and perfectionism (Gramaglia et al., 2017; Parra-Fernández et al., 2018). Conversely, higher BMI individuals may prioritize practical eating behaviors over rigid dietary patterns due to physical health concerns or social factors (Dunn & Bratman, 2016). Further research is needed to explore these potential explanations in different cultural contexts.

Furthermore, in the correlational analysis of participants with ON tendency, mean ORTO-11 scores, and TEMS sub-dimensions, a positive and statistically significant relationship was found between participants’ mean ORTO-11 scores and “weight control motivation”, indicating that as the weight control motivation increased, ON tendency decreased (*p* < 0.05). Conversely, a study by Foyster et al. (2023) found a strong positive correlation between weight control motivation and higher ON tendency (*p* < 0.001). This contradiction may be due to differences in the samples. University students may be more likely to associate health motivation with other factors such as academic performance, stress management, or social acceptance. In addition, health motivation in this age group may be shaped differently by the influence of social media and dietary trends. It is recommended that future studies compare different demographic groups to further investigate the context-specific effects of health motivation on orthorexia nervosa. In a study conducted with athletes, it was found that there was no correlation between body weight control motivation and ON tendency (*p* > 0.05). Normally, orthorectic behaviors are not a healthy way of maintain optimal nutritional behaviors, and, in the case of athletes, the authors reported that participants were more aware of ON (World Health Organization, 2005). Maintaining balanced nutrition and an healthy lifestyle is required for staying within the ideal BMI ranges recommended by the World Health Organization (18.5–24.9 kg/m^2^) (WHO).

In the effect analysis of the TEMS sub-dimensions on ORTO-11 scale, “health” was found to be significantly and negatively associated with ON tendency (higher mean ORTO-11 scores) of participants (*p* < 0.05). In contrast, Foyster et al. (2023) found that an eating motivation such as “health”, which is considered to be a basic component of orthorexia, was positively associated with ON tendency. In different cultural or demographic groups, the meaning or impact of health motivation may be different. In addition, “traditional eating” sub-dimension of TEMS had a negative and statistically significant effect on mean ORTO-11 scores of participants with ON tendency (*p* < 0.05). In other words, having motivation for traditional eating habits had an increasing effect on ON tendency.

Orthorexics often worry when their food comes from sources of uncertain quality, may get stuck and prolong the preparation of their meals, may follow inflexible self-imposed rules, and may tend to express feelings of superiority towards other people who do not share their eating habits (Gkiouleka et al., 2022). They may, therefore, move away from traditional eating habits or feel guilty after eating these foods.

The observed negative relationship between traditional eating motivation and orthorexia nervosa (ON) tendencies is intriguing and warrants deeper exploration. One possible explanation is that traditional eating habits, which often emphasize shared meals, cultural practices, and culinary diversity, may counteract the rigid and restrictive patterns associated with orthorexic behaviors. Cultural factors likely play a pivotal role in this relationship. Traditional diets, rooted in long-standing customs, are often more flexible and inclusive. For example, the Mediterranean diet, which incorporates a variety of foods and social eating practices, has been associated with improved mental well-being and reduced disordered eating behaviors (Bach-Faig et al., 2011). Similarly, research suggests that cultural practices that celebrate food as part of communal and festive activities may buffer individuals against the isolating and obsessive tendencies of orthorexia (Varga et al., 2014). Further studies are needed to investigate whether the inherent flexibility and cultural richness of traditional diets act as protective factors against ON. These findings could offer insights into how fostering traditional food practices might support healthier relationships with food.

The major strength of this study is that it is the first study conducted with TEMS in Turkey to determine whether the factors that influence eating motivation have an effect on ON. This study’s novel use of the TEMS scale in Turkey provides valuable insights into eating motivations and their relationship with orthorexic tendencies, particularly in university settings. The findings can inform practical interventions, such as awareness programs that promote balanced eating behaviors and traditional food practices, as well as tailored counseling services for students at risk of ON. Additionally, TEMS can be utilized for long-term monitoring to assess the effectiveness of preventive strategies. By emphasizing the practical implications, this research contributes to both academic understanding and the development of culturally sensitive approaches to support healthy eating behaviors.

One limitation of this study is the gender imbalance in the sample, with 84.4% of participants being female. This disproportion may limit the generalizability of the findings, particularly to male populations, as previous research suggests gender differences in eating behaviors and motivations (Wardle et al., 2006; Griffiths et al., 2016). Future studies should aim to recruit a more gender-balanced sample to ensure that the findings reflect the broader population. Additionally, conducting separate analyses for male and female participants could help to identify potential gender-specific patterns in eating motivations and orthorexic tendencies. Such approaches would enhance the robustness and applicability of the research, contributing to a more comprehensive understanding of these phenomena. The risk of orthorexia, not just orthorexia nervosa, is assessed by the ORTO-11 scale used in this study. Furthermore, this study did not discuss in detail the distinction between healthy orthorexia and orthorexia nervosa. This is considered a limitation. It should be considered when interpreting the results. Although the quantitative variables explained 6.7% of the variance in orthorexia nervosa (R^2^ = 0.067), the effect size is relatively small, which represents a limitation of the study. This suggests that other unexamined factors may contribute to ON, and future research with larger and more diverse samples is needed to better understand these relationships. The cross-sectional design of this study limits the ability to assess causal relationships or observe changes over time. It only provides a snapshot of the data, which restricts the generalizability of the findings. Future research should consider longitudinal designs to track changes in eating behaviors and orthorexic tendencies over time, allowing for more robust conclusions. The use of self-reported BMI is another limitation as participants’ self-assessments may not accurately reflect their actual BMI due to bias or lack of awareness. Future studies could employ more objective measurement techniques, such as clinical BMI assessments or additional biometric data, to improve accuracy. These limitations should be acknowledged when interpreting the results, and addressing them in future research will strengthen the overall findings.

The findings of this study can inform intervention programs aimed at reducing orthorexic tendencies among university students. Awareness campaigns promoting balanced eating behaviors, as well as workshops focused on flexible dietary practices, could help to reduce the rigid thinking associated with ON. Additionally, university counseling services could integrate these findings into individualized support programs. For future research, studies could explore the effectiveness of specific intervention strategies, such as mindfulness-based approaches or group counseling, in addressing ON tendencies. Expanding the sample to include different universities or cultural contexts would also provide valuable insights into the broader applicability of these interventions.

## 5. Conclusions

In conclusion, understanding the motivational factors underlying eating behaviors in younger populations is crucial for addressing orthorexic tendencies (ON). The findings suggest that cultural values, health-related motivations, and social influences can contribute to the development of ON. For example, rigid adherence to healthy eating can be influenced by the pressure to conform to societal ideals of health and body image. These factors may lead to an unhealthy preoccupation with food quality and control, which can manifest as ON. Future intervention programs should focus on promoting flexibility in eating behaviors and addressing the underlying motivations for restrictive eating. This approach will help to reduce the prevalence of ON and encourage healthier, more balanced dietary habits in young people.

Understanding motivational factors behind eating is crucial, but the link between these motivations and ON should be explored in more detail. For instance, motivations like the desire for health or body image may influence eating behaviors by leading individuals to adopt restrictive diets. Additionally, these motivations can affect treatment outcomes as individuals with stronger health-driven motivations might struggle with flexible eating approaches in therapy.

Environmental factors, peer pressure, and misinformation contribute to ON. Social media and societal health ideals create unrealistic expectations about food and body image, prompting individuals to adopt rigid dietary patterns to meet perceived health standards. Peer pressure, especially in university settings, can reinforce restrictive eating behaviors. Misinformation about nutrition can mislead individuals into overly cautious or restrictive eating, often leading to ON. Understanding these contributions is key to designing effective interventions for ON.

Organizing training on balanced nutrition, eating disorders, and healthy lifestyles is a valid and important recommendation. However, to enhance its practicality, it would be beneficial to provide more specificity regarding the type of training and content to be prioritized. For instance, it could be helpful to clarify whether the training should take the form of workshops, seminars, or peer-led discussions. Additionally, focusing on evidence-based guidelines and actionable strategies would ensure the training is both informative and effective. Providing these concrete details would make the recommendation more actionable and relevant to the target audience.

## Figures and Tables

**Table 1:** Distribution of descriptive findings and quantitative variables.

Variables		n	%
Sex			
Female		351	84.4
Male		65	15.6
Martial status			
Married		34	8.2
Single		382	91.8
Level of education			
Associate Degree		18	4.3
License		333	80
Postgraduate		65	15.6
Weekly physical activity status			
Less than 150 min per week		191	45.9
Between 150 and 299 min per week		160	38.5
300 min and above per week		65	15.6
Satisfaction status with current body weight			
Yes		185	44.5
No		231	55.5
Age (year)	Min–Max	17–44 22.3 ± 4.41	
	Mean. ± SD		
Weight (kg)		40–148 63.24 ± 14.87	
Height (cm)		149–196 1.66 ± 0.07	
BMI (kg/m^2^)		15.1–49.5 22.74 ± 4.54	
ORTO-11		27.55 ± 4.71	

**Table 2:** Comparison of the ORTO-11 by descriptive variables.

	Group				
	Tendency to ON	No Tendency to ON	Total	X^2^	*p*
Sex					
Female	168 (81.6%)	183 (87.1%)	351 (84.4%)	2.464	0.076 *
Male	38 (18.4%)	27 (12.9%)	65 (15.6%)		
Martial status					
Married	14 (6.8%)	20 (9.5%)	34 (8.2%)	1.031	0.202 *
Single	192 (93.2%)	190 (90.5%)	382 (91.8%)		
Level of education					
Associate Degree	5 (2.4%)	13 (6.2%)	18 (4.3%)	4.635	0.099
Licence	172 (83.5%)	161 (76.7%)	333 (80%)		
Postgraduate	29 (14.1%)	36 (17.1%)	65 (15.6%)		
Presence of chronic disease					
Yes	23 (11.2%)	20 (9.5%)	43 (10.3%)	0.302	0.349 *
No	183 (88.8%)	190 (90.5%)	373 (89.7%)		
Smoking status					
Yes	48 (23.3%)	50 (23.8%)	98 (23.6%)	0.015	0.497 *
No	158 (76.7%)	160 (76.2%)	318 (76.4%)		
Alcohol use status					
Yes	10 (4.9%)	9 (4.3%)	19 (4.6%)	0.077	0.483 *
No	196 (95.1%)	201 (95.7%)	397 (95.4%)		
Weekly physical activity status					
Less than 150 min per week	98 (47.6%)	93 (44.3%)	191 (45.9%)	1.979	0.372
Between 150 and 299 min per week	81 (39.3%)	79 (37.6%)	160 (38.5%)		
300 min and above per week	27 (13.1%)	38 (18.1%)	65 (15.6%)		
Satisfaction status with current body weight					
Yes	112 (54.4%)	73a (34.8%)	185 (44.5%)	16.189	<0.001 *
No	94 (45.6%)	137_b_ (65.2%)	231 (55.5%)		

Chi-square test, * Fisher’s Exact test.

**Table 3:** Relational analysis of quantitative variables with TEMS sub-dimensions.

		Liking	Habits	Need and Hunger	Health	Convenience	Pleasure	Traditional Eating	Natural Concern	Sociability	Price	Visual Appeal	Weight Control	Affect Regulation	Social Norms	Social Image
**Age**	r	0.028	−0.021	0.017	0.046	0.032	0.027	−0.014	0.035	0.058	0.002	−0.048	−0.008	−0.018	0.014	−0.061
	*p*	0.566	0.673	0.726	0.345	0.515	0.588	0.774	0.474	0.240	0.962	0.329	0.864	0.721	0.777	0.215
**Body weight**	r	−0.089	−0.042	−0.038	0.059	−0.064	−0.038	0.022	−0.037	0.079	0.057	0.017	**0.153**	**0.180**	0.073	**0.108**
	*p*	0.071	0.397	0.442	0.233	0.193	0.435	0.652	0.451	0.107	0.245	0.723	**0.002**	**0.000**	0.140	**0.028**
**Height**	r	−0.016	0.044	0.084	**0.120**	0.019	0.036	**0.102**	0.046	0.062	0.072	0.012	**0.121**	0.011	0.039	0.046
	*p*	0.746	0.371	0.087	**0.014**	0.694	0.466	**0.038**	0.354	0.207	0.145	0.805	**0.013**	0.830	0.426	0.351
**BMI**	r	−0.091	−0.065	−0.080	0.008	−0.087	−0.043	−0.013	−0.061	0.065	0.025	0.005	**0.114**	**0.203**	0.077	0.089
	*p*	0.063	0.186	0.103	0.866	0.076	0.376	0.791	0.214	0.189	0.608	0.913	**0.020**	**0.000**	0.118	0.068

**Table 4:** Relational analysis of mean ORTO-11 scores of ON-tendent participants and TEMS dimensions.

		Liking	Habits	Need and Hunger	Health	Convenience	Pleasure	Traditional Eating	Natural Concern	Sociability	Price	Visual Appeal	Weight Control	Affect Regulation	Social Norms	Social Image
**ON-tendent**	r	−0.035	−0.126	−0.121	**0.171**	0.033	−0.029	**−0.143**	0.131	0.099	0.013	−0.024	**0.160**	−0.040	0.056	0.018
	*p*	0.616	0.071	0.083	**0.014**	0.640	0.679	**0.041**	0.061	0.157	0.850	0.728	**0.022**	0.568	0.423	0.796

Spearman correlation test.

**Table 5:** Impact analysis of descriptive quantitative variables and TEMS sub-dimensions in relation to mean ORTO-11 scores of ON-tendent participants.

	F	*p*	R^2^	B	*t*	*p*
(Constant)	4.832	0.003	0.067	19.410	15.487	0.000
Age				0.060	1.325	0.187
Weight				−0.023	−0.919	0.359
BMI				0.195	2.291	0.023
(Constant)	5.788	0.001	0.079	23.757	46.709	0.000
Health				0.404	2.030	0.044
Traditional Eating				−0.507	−3.730	0.000
**Weight Control**				0.096	0.504	0.615

Multiple linear regression test.

## Data Availability

The data that support the findings of this study will be made available by the corresponding author upon reasonable request. Data are not publicly available due to privacy and ethical concerns.
